# Gait Phase Recognition in Multi-Task Scenarios Based on sEMG Signals

**DOI:** 10.3390/bios15050305

**Published:** 2025-05-10

**Authors:** Xin Shi, Xiaheng Zhang, Pengjie Qin, Liangwen Huang, Yaqin Zhu, Zixiang Yang

**Affiliations:** 1 School of Automation, Chongqing University, Chongqing 400044, China; 13238642690@163.com (X.Z.); 202313021026@stu.cqu.edu.cn (L.H.); 202213131085t@stu.cqu.edu.cn (Y.Z.); 13987145770@163.com (Z.Y.); 2 Shenzhen Insitute of Advanced Technology, Chinese Academy of Sciences, Shenzhen 518000, China

**Keywords:** gait phase recognition, sEMG, fApEn, feature extraction, CNN

## Abstract

In the human–exoskeleton interaction process, accurately recognizing gait phases is crucial for effectively assessing the assistance provided by the exoskeleton. However, due to the similarity in muscle activation patterns between adjacent gait phases, the recognition accuracy is often low, which can easily lead to confusion in surface electromyography (sEMG) feature extraction. This paper proposes a real-time recognition method based on multi-scale fuzzy approximate root mean entropy (MFAREn) and an Efficient Multi-Scale Attention Convolutional Neural Network (EMACNN), building upon the concept of fuzzy approximate entropy. MFAREn is used to extract the dynamic complexity and energy intensity features of sEMG signals, serving as the input matrix for EMACNN to achieve fast and accurate gait phase recognition. This study collected sEMG signals from 10 subjects performing continuous lower limb gait movements in five common motion scenarios for experimental validation. The results show that the proposed method achieves an average recognition accuracy of 95.72%, outperforming the other comparison methods. The method proposed in this paper is significantly different compared to other methods (*p* < 0.001). Notably, the recognition accuracy for walking in level walking, stairs ascending, and ramp ascending exceeds 95.5%. This method demonstrates a high recognition accuracy, enabling sEMG-based gait phase recognition and meeting the requirements for effective human–exoskeleton interaction.

## 1. Introduction

Surface electromyogram (sEMG) signals have advantages such as rich information content [1], motion extraction capability, and non-invasive acquisition [2]. Electromyography-based intention recognition technology is widely applied in human–machine interactive control systems with neural feedback, such as lower-limb power-assisted exoskeletons, intelligent prosthetics, and rehabilitation robots. Among them, lower-limb exoskeletons can enhance the wearer’s load-bearing capacity and reduce physical exertion, and are widely used in fields such as logistics sorting, instrument inspection, and terrain exploration. Currently, the main challenge in the research on electromyography-based lower-limb exoskeleton assistance lies in achieving seamless human–machine interaction and control by sensing human intentions. Considering the complexity of user interaction, exoskeletons must assist rather than impede users [3]. If the movement intention is not accurately detected, the exoskeleton may misinterpret it, causing poor coordination and potential harm. Continuous motion requires timely adaptation to changing gait patterns, demanding high interaction accuracy. Abnormal gait can reduce mobility, increase energy use, and lead to negative effects. Thus, identifying gait phases based on both legs is essential, and fast, accurate timing is key to effective human-exoskeleton collaboration [4].

Currently, commonly used gait phase recognition algorithms include machine learning [5,6,7] and deep learning [8,9,10,11]. Traditional machine learning relies on handcrafted features, which may struggle with the nonlinear and complex nature of sEMG signals, leading to limited robustness and accuracy. In contrast, deep learning offers powerful nonlinear mapping and automatic feature extraction, enabling better adaptability and higher recognition accuracy through multi-layered architectures.

The sensors commonly used for gait phase recognition include Inertial Measurement Units (IMU) [12,13,14,15,16,17,18], EEG, sEMG [19,20,21], and others. To extract comprehensive features, some studies have employed multi-sensor fusion methods for gait phase recognition, including combinations such as IMU with sEMG [22], plantar pressure with acceleration [23], IMU, sEMG, and plantar pressure [10], and pressure sensors with IMU [24]. Although IMU sensors achieve high accuracy in motion state recognition due to their precise posture sensing, their reliance on mechanical feedback often results in delayed responses, limiting their ability to predict upcoming motion transitions. While multimodal fusion enhances complementary information extraction, it also poses challenges such as inconsistent dimensions, sampling rates, and response timings. The sEMG signals have gradually emerged as an important means of characterizing human movement intention because they precede human movement and can provide information about neuromuscular activity [25]. It also has characteristics such as non-invasive acquisition and low latency. The sEMG signals are generated 30–260 milliseconds before human motion, meaning that they can be used to predict movement [26]. Therefore, the prediction of human motion can be realized via feature extraction and classification technology [27]. This study aims to investigate gait phase recognition based on sEMG signals.

The sEMG signals are weak, nonlinear physiological signals with chaotic and dynamically time-varying characteristics. Additionally, they are highly susceptible to noise during acquisition, making direct and rapid gait phase recognition challenging. To address these issues, this paper proposes the MFAREn-EMACNN lower limb gait phase recognition method, which decodes the human body’s motion intention and recognizes gait phases from sEMG signals. The objectives and contributions of this study are mainly threefold:

1. Based on the theoretical foundation of fuzzy approximate entropy, multi-scale analysis and RMS are introduced for feature extraction, constructing multi-channel electromyographic features. It can effectively capture the dynamic features across multiple scales and significantly distinguish between resting and active states of the sEMG signals.
2. Construct the EMACNN model and integrate sEMG entropy features to achieve gait phase recognition.
3. Identify gait phases solely from sEMG signals and provide experimental evaluation to demonstrate the effectiveness and generalization of the proposed model.

## 2. Motion Data Acquisition and Preprocessing

### 2.1. Data Acquisition

**Figure 1:**
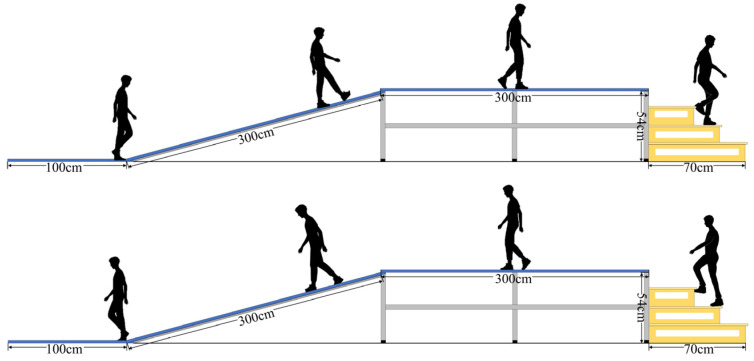
Experimental scene diagram.

The daily movements of human beings include walking, running, ascending and descending stairs, traversing uphill and downhill slopes [28,29], etc. The schematic diagram of the experimental environment constructed in this paper is depicted in Figure 1. From left to right, it features flat ground, a ramp with a 20° slope, another section of flat ground, and staircase scenes, accommodating five movement patterns of daily life: level walking (LW), stairs ascending (SA), stairs descending (SD), ramp ascending (RA), and ramp descending (RD).

**Figure 2:**
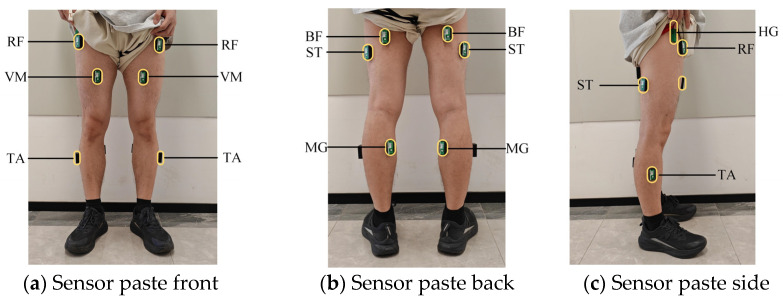
Myoelectric sensor pasting diagram.

The surface electromyography sensors used in this study are wireless sensors manufactured by Biometrics Ltd. Newport, UK. The model of the angle sensor and the foot pressure sensor is FS-INS-3Z-V2. The sensor circuit is mainly composed of electrodes, amplification circuits, and filters. Reusable integral dry electrodes are used to convert the bioelectric signals generated by muscle activity into electrical currents. The amplification circuit enhances the weak signal amplitude collected by the electrodes for more accurate measurement and analysis. Filters are used to remove common interferences, such as power frequency signal interference and baseline drift, during the signal acquisition process. Twelve sEMG sensors, each with a sampling rate of 1000 Hz, are placed on the right and left legs at the rectus femoris muscle (RF), vastus medialis muscle (VM), semitendinosus muscle (ST), biceps femoris muscle (BF), tibialis anterior (TA), and medial gastrocnemius (MG). A hip goniometer (HG) is positioned at the hip joint of the right leg, and a plantar pressure sensor is placed inside the shoe. The sensors are affixed at the positions illustrated in Figure 2. During sensor placement, the subjects were instructed to contract their muscles, and each muscle was identified through palpation of the muscle belly to ensure accurate positioning. The sEMG signal electrodes were applied according to the SENIAM guidelines, ensuring consistency in placement and the reliability of the data acquisition process, and the subjects completed five movements in the experimental scenarios: level walking (LW), stairs ascending (SA), stairs descending (SD), ramp ascending (RA), and ramp descending (RD). Consequently, the corresponding motion information could be captured.

A total of 12 healthy male subjects were recruited for the related experiments. They ranged in age from 21 to 31 years old, with heights between 1.68 and 1.82 m and weights between 61.5 and 78.2 kg. All the subjects were informed about the experimental content and signed an informed consent form prior to the commencement of the experiment. The experiment was conducted in a quiet environment with minimal electromagnetic interference. Before the data collection, the target areas on the participants’ lower limbs were cleaned with alcohol to reduce skin impurities and interference. Specialized EMG electrode patches were used to securely attach the sensors to the corresponding muscle areas, preventing them from detaching during the data collection. To effectively eliminate the impact of muscle fatigue on the signal quality, each participant was given adequate rest before the data collection. The data were collected in 1 min intervals between each group, and after every 20 data sets, a 10 min rest period was provided. This approach ensured that the collected data accurately reflected the natural muscle activity while significantly reducing the interference from fatigue.

### 2.2. Data Preprocessing

The surface electromyographic signals are potentials captured on the skin during the contraction and relaxation of human muscle fibers. These signals may be affected by various factors, such as the relative displacement between the electrodes and the skin. To accurately understand the movement intention from these measurements, it is necessary to eliminate possible noise. The sEMG signals acquired for each walking experiment first underwent processing through a Butterworth 4th-order bandpass filter with a low-frequency cutoff of 20 Hz and a high-frequency cutoff of 200 Hz, as well as a 60 Hz notch filter [30], which can eliminate most of the intrinsic device noise and motion artifacts.

**Figure 3:**
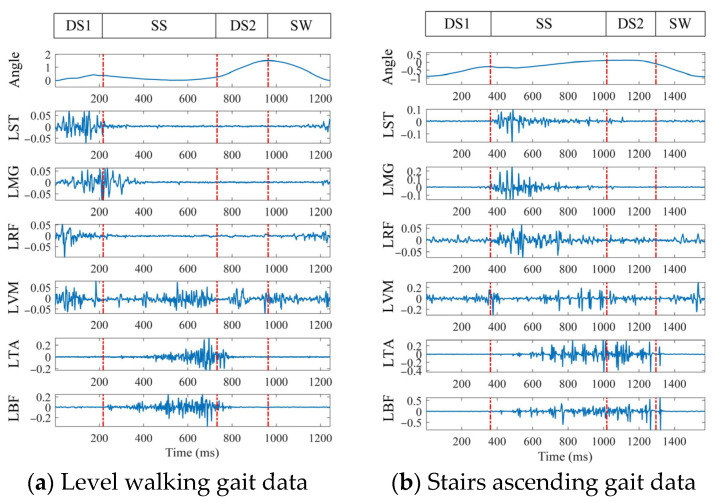
Level walking and stairs ascending gait phase data diagram.

After the data acquisition and preprocessing, a gait motion information graph was plotted and is shown in Figure 3. A gait cycle refers to the time it takes for the human body to complete one full gait, which is the movement process from when one foot leaves the ground to when the same foot leaves the ground again. Its sub-stages can be divided into four phases: the first double support phase (DS1), the single support phase (SS), the second double support phase (DS2), and the swing phase (SW) [31]. The gait phase segmentation is based on the variation in the hip joint angle. Before collecting continuous motion data, the hip joint angle variation trend for each subject during different gait phases was recorded. Then, by combining the maximum and minimum values of the joint angle within a complete gait cycle, the stance phase and swing phase were divided into early stance, late stance, early swing, and late swing phases.

## 3. Multi-Task Scene Gait Phase Recognition Model Based on sEMG

**Figure 4:**
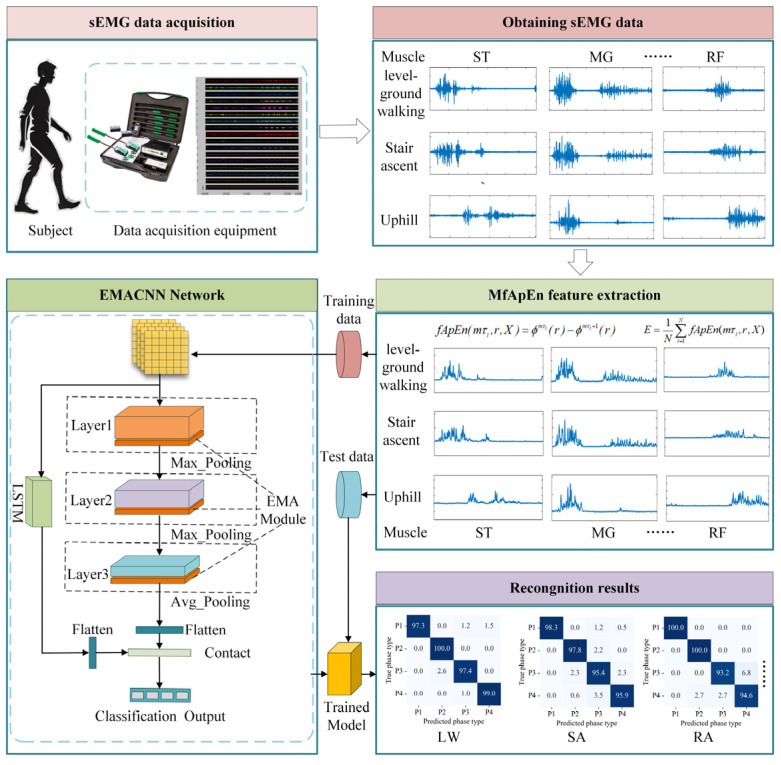
Structure of gait phase recognition model implementation.

In this paper, a framework based on the sEMG signals, feature extraction, and a recognition network is constructed, as shown in Figure 4. The specific steps are as follows:

### 3.1. Gait Phase Feature Extraction Based on MFAREn

The collected sEMG signals were more complex compared to the IMU signals, and there were redundant signals in the raw sEMG signals collected [32]. On the one hand, they contain motion steady-state information; on the other hand, they also contain transient information. The feature extraction methods used in the previous studies often rely on time-domain, frequency-domain, and time–frequency domain methods to analyze the similarity of sEMG signals between adjacent phases. However, time-domain and frequency-domain methods neglect the detailed fluctuation information of sEMG signals, while time–frequency domain methods consume a lot of time for signal decomposition, which cannot meet the timeliness requirements of feature extraction. Therefore, in this paper, fuzzy approximate entropy (fApEn) [33] is used for feature extraction of multi-channel lower limb sEMG signals. fApEn is an effective and stable algorithm for evaluating the complexity of physiological signals, and due to the nonlinear and temporal nature of sEMG signals, fApEn can be used to characterize the change rule of muscle force generation.

By incorporating fuzzy rules into approximate entropy, dynamic and complex representations can be achieved. The detailed calculation steps are as follows:

If a time sequence $N=N_{1},N_{2},\ldots ,N_{L}$ is given, where L is the length of the sequence N, and the sequence is sequentially composed into vectors of m-dimensional $X_{i}^{m}$, the vector sequence takes a form similar to the definition of approximate entropy as follows:

For a time series $N=N_{1},N_{2},\ldots ,N_{L}$ containing N data points, when considering the same time sequence T, the sEMG signals’ vector sequence takes a form similar to the definition of approximate entropy:

(1) $$X_{i}^{m}=\{u(i),u(i+1),\ldots ,u(i+m-1)\}-u_{0}(i),i=1,\ldots ,N-m+1$$

To enhance the signal features, where $X_{i}^{m}$ obtains the baseline value $u_{0}(i)$ by eliminating the baseline, $u_{0}(i)$ can be calculated as shown in Equation (2).

(2) $$u_{0}(i)=\frac{1}{m}\sum_{j=0}^{m-1}u(i+j)$$

The fuzzy approximate entropy of an N-dimensional signal is then defined as:

(3) $$fApEn(m,r,X)=\phi^{m}(r)-\phi^{m+1}(r)$$

Equation $\phi^{m}(r)$ is calculated as follows:

(4) $$\phi^{m}(r)={\left[N-(m-1)\tau \right]}^{-1}\sum_{i=1}^{N-(m-1)\tau }\ln(C_{i}^{m}(r))$$

(5) $$C_{i}^{m}(r)=\frac{1}{N-(m-1)\tau }\sum_{j=1}^{N-(m-1)\tau }D_{ij}^{m}$$

where N is the number of time points, m is the pattern length, τ is the delay time parameter, and the fuzzy affiliation function is used to determine the $D_{ij}^{m}$ in the computation of $C_{i}^{m}(r)$, where $D_{ij}^{m}$ is calculated as follows:

(6) $$D_{ij}^{m}=u(d_{ij}^{m},r)$$

$d_{ij}^{m}$ is used to define the distance between the sequences $X_{i}^{m}$ and $X_{j}^{m}$ (m dimensional pattern vectors):

(7) $$d_{ij}^{m}=d[X_{i}^{m},X_{j}^{m}]=\underset{k\in (0,m-1)}{\max}\left|u(i+k)-u_{0}(i)-(u(j+k)-u_{0}(j))\right|$$

where i,j=1,2,…,N−m+1 Fuzzy affiliation functions can be used to describe the similarity between two vectors. Generally, functions such as the Gaussian function, Gaussian kernel function, and Sigmoid function can be chosen to describe the similarity between two vectors. In this study, the Gaussian kernel function is chosen to serve as the fuzzy affiliation function for the fuzzy approximate entropy calculation. The function $u(d_{ij}^{m},r)$ is defined as follows:

(8) $$u(d_{ij}^{m},r)=\exp\left(-\frac{d_{ij}^{2}}{2r^{2}}\right)$$

where r is a predefined tolerance value, usually defined as:

(9) r=k⋅std(X)

where k is a constant, k>0, and std(⋅) denotes the standard deviation of the signal.

Finally, for a finite dataset, the entropy of a segment of time-series signals can be calculated using $fApEn(m,r,X)=\phi^{m}(r)-\phi^{m+1}(r)$.

Through experiments, it is found that modal aliasing is easy to occur between the gait phases, and a high degree of similarity between neighboring phases occurs due to the similarity of the muscle force patterns, which can easily lead to a reduction in the accuracy of the final phase recognition if no significant sEMG features can be extracted, so a feature that can reflect the degree of change of the sEMG signals in gait phases is needed to be used as an input value for the phase recognition model.

While fApEn can be used to measure the complexity of nonlinear signals, it is still to be enhanced for the capture of local features. Therefore, in this paper, we introduce the multi-scale factor, which can capture the dynamic features of the sEMG signals in different time scales, and then comprehensively characterize the non-stationarity of complex time series.

Calculating eigenvalues at different scales: the embedding matrix is first generated based on the embedding dimension m and the time series {u(i)}:

(10) $$X_{i}^{m}=\{u(i),u(i+1),\ldots ,u(i+m-1)\}-u_{0}(i),i=1,\ldots ,N-m+1$$

(11) $$Y_{i}^{m}=\{u(i),u(i+1),\ldots ,u(i+m)\}-u_{0}(i),i=1,\ldots ,N-m$$

Generate a multi-scale subsequence, define the scale arrays τ, $\tau_{i}<m+1,i=1,2,\ldots ,N$, and for each scale, the entropy value will be calculated based on a different embedding dimension $m\cdot \tau_{i}$.

The multi-scale entropy calculation: at each scale, the entropy value under the embedding dimension $m\cdot \tau_{i}$ can be obtained by calculating the entropy value according to $fApEn(m,r,X)=\phi^{m}(r)-\phi^{m+1}(r)$ separately:

(12) $$E_{X_{i}^{m}}=fApEn(m\tau_{i},r,X)=\phi^{m\tau_{i}}(r)-\phi^{m\tau_{i}+1}(r)$$

(13) $$E_{Y_{i}^{m}}=fApEn(m\tau_{i},r,Y)=\phi^{m\tau_{i}}(r)-\phi^{m\tau_{i}+1}(r)$$

Combining the Kullback–Leibler Divergence measures the difference between two or more probability distributions, reflecting the changes in the distribution of the signal at multiple scales. Then, by cumulatively averaging the entropy values E under all scale factors, MfApEn can be obtained, as indicated in Equation (14).

(14) $$MfApEn=-\frac{1}{\tau_{N}}\sum_{s}\sum_{i}E_{Y_{i}^{m}}.\log(\frac{E_{Y_{i}^{m}}+\varepsilon }{E_{Y_{i}^{m}}+\varepsilon })$$

where $\varepsilon =10^{-6}$ is the smoothing term, preventing division by 0 errors in logarithmic calculations, and $\tau_{N}$ is the length of the set of scales.

The RMS can be used to characterize the statistical characteristics of the sEMG signals, reflecting the contraction characteristics of the muscle through the amplitude and taking into account the non-linear characteristics. It can characterize the dynamic range of the signal.

(15) $$RMS=\sqrt{\frac{1}{N}\sum_{i=1}^{N}x_{i}^{2}}$$

Adding the multi-scale fuzzy approximation entropy to the RMS gives *MFAREn*, which can reflect the dynamic complexity of the sEMG signals as well as highlight the energy characteristics of the sEMG signals.

(16) MFAREn=MfApEn(mτ,r,i)+RMS(i)

Applying different subsequence lengths and feature calculation methods to the same time series captures dynamic characteristics at multiple scales. This approach, known as multi-scale fuzzy approximate root mean entropy (MFAREn), enhances the identification of nonlinear features in EMG signals, facilitating the analysis of multi-channel and complex time series.

### 3.2. Efficient Multi-Scale Attention Convolutional Neural Network

Since neural networks have strong learning capabilities for nonlinear data, they are suitable for handling classification problems based on the sEMG signals, which is a type of nonlinear time-varying sequence. In the complex process of lower limb movement classification, neural network models need to iteratively learn during training, resulting in slow convergence and a tendency to fall into local optima [34]. The EMACNN discussed in this paper primarily consists of convolutions, batch normalization, average pooling, EMA (Efficient multi-scale attention) [35], and fully connected outputs. It comprises three layers, each with its own convolution kernel, forming a multichannel attention convolution. The EMA focuses on preserving information from each channel while reducing the computational overhead by reshaping certain channels into the batch dimension and grouping channel dimensions into multiple sub-features. This ensures a uniform distribution of sEMG features within each feature group, encodes cross-channel information to adjust the importance of different channels, and aggregates cross-spatial information through entropy in different spatial dimensions to enhance the model’s feature-learning capability. The multiscale CNN module employs a CNN to learn multiscale spatial features from sEMG signal samples.

**Figure 5:**
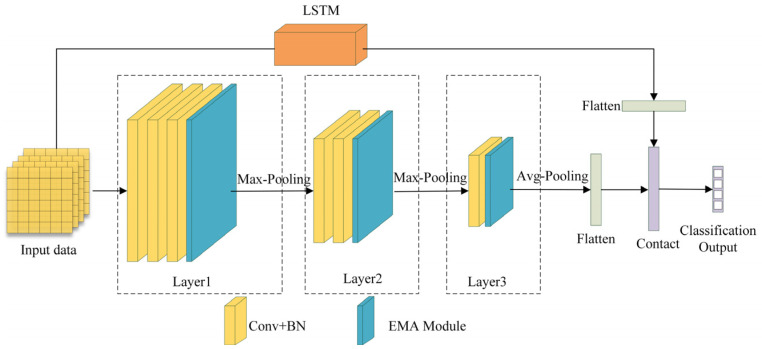
EMACNN model structure.

In the EMACNN model, the multi-scale convolutional layer employs 5 × 5, 3 × 3, and 1 × 1 convolutional kernels to extract the scale features from the input data. All the convolutional layers use a 2 × 2 pooling kernel, and BatchNorm is applied to normalize the features and prevent overfitting. The overall neural network structure can be divided into three layers; the neural network structure is illustrated in Figure 5.

(1) Input Layer: The input layer of the EMACNN model computes the 12-channel lower limb sEMG signals using MFAREn, resulting in a shape of 1 × 12 × 91 (the overlapping sliding window method is used, with 100 ms of data, a window length of 10, and a window overlap of 1). Here, 1 represents the number of signal components, 12 represents the signal channels, and 91 represents the 91 feature values.

(2) First Layer (Layer1): This layer consists mainly of three 2-dimensional convolution kernels and an EMA module. To fully learn the basic features of the input data, each convolution block has a kernel size of 3 × 3. The EMA module enriches the myoelectric feature aggregation by providing a cross-space information aggregation method in different spatial dimensional directions. An EMA generates a spatial attention map, and the final EMA output is the same size as the input, allowing it to be effectively stacked into the CNN architecture.

(3) Second Layer (Layer2): This layer consists mainly of two 2D convolutional blocks and an EMA module, designed to learn deeper sEMG features in the output feature map of the first layer after maximum pooling.

(4) Third Layer(Layer3): This layer primarily consists of a 2-dimensional convolutional block and an EMA module, which work together to aggregate the previously learned features. Firstly, maximum pooling is applied, followed by attention focusing through the EMA, enabling efficient aggregation of deeper sEMG features.

(5) LSTM Layer: This layer operates in parallel with the convolutional layers to extract the temporal features of the sEMG signals. The final outputs of the temporal features and the convolutional results are concatenated into a vector matrix.

(6) Output Layer: This layer consists of fully connected layers to complete the classification output based on the feature information.

In this paper, the cross-entropy loss function is used as the cost function for model training. Assuming that each input contains N samples and the data are independently and identically distributed, the likelihood function can be obtained as shown in Equation (17). Taking the logarithm of the likelihood function, simplifying it, and then computing its negative yields the cross-entropy loss as shown in Equation (18).

(17) $$L\left(x,y\right)=\prod_{i=1}^{N}{\left[h_{\theta }(x_{i})\right]}^{\left(y_{i}\right)}{\left[1-h_{\theta }(x_{i})\right]}^{\left(1-y_{i}\right)}$$

(18) $$J(\omega ,b)=-\sum_{i=1}^{N}\left[y_{i}\log(h_{\theta }(x_{i}))+(1-y_{i})\log(1-h_{\theta }(x_{i}))\right]$$

In this paper, the Adam algorithm is used as the optimizer, and the learning rate is set to a fixed-step decay. The training epoch and batch size are set according to the number of samples in the datasets to ensure model convergence [36].

### 3.3. Evaluation Metrics

In this paper, we use accuracy, recall, precision, and F1-score to evaluate the classification performance of the lower limb gait phase recognition method. Defining true positive (*TP*) as the number of samples correctly predicted as the positive class, true negative (*TN*) as the number of samples correctly predicted as the negative class, false positive (*FP*) as the number of samples incorrectly predicted as the positive class, and false negative (*FN*) as the number of samples incorrectly predicted the negative class.

(1) Accuracy

The accuracy rate indicates the ratio of the number of correctly predicted samples to the total number of samples. It reflects the overall performance of the model and is calculated using the following formula:

(19) $$Accuracy=\frac{TP+TN}{TP+TN+FP+FN}$$

(2) Precision

Precision indicates the proportion of samples in the classification model’s prediction of the current phase that are actually in that phase in the real data. It can be calculated using the following formula:

(20) $$Precision=\frac{TP}{TP+FP}$$

(3) Recall rate

Recall represents the ratio of the number of samples in the classification model that correctly identify the gait phase to the total number of samples that are actually in that phase. It can be calculated using the following formula.

(21) $$Recall=\frac{TP}{TP+FN}$$

(4) F1-score

The F1-score is the harmonic mean of precision and recall. The higher the F1-score, the more accurate the prediction result. It can be calculated using the following formula:

(22) $$F1-score=\frac{2\cdot Precision\cdot Recall}{Precision+Recal}$$

(5) Average time cost

The real-time performance of the model is evaluated by calculating the recognition time of each sample.

(23) $$T=\frac{1}{n}\sum_{i=1}^{n}t_{i}$$

Among them, $t_{i}$ represents the recognition time of the i sample, and n is the number of recognized samples.

## 4. Experimental Validation

In this study, we used a laptop with an Intel i5-12400F @2.5GHz CPU and an NVIDIA RTX 3060 GPU as the hardware environment. The software environment consists of Python with PyTorch 2.2.2. A 5-fold cross-validation strategy was employed during training. Given that the algorithmic models are intended for exoskeleton hardware, all the model training was conducted on the GPU, while data testing was performed on the CPU. For each type of movement pattern, 3200 samples with a data length of 100 ms were taken. With a total of 12 subjects, this resulted in 38,400 data samples per movement type. For five types of movement patterns, the total number of data samples was 192,000. In this study, we utilized the first 80% of the data as the training set and the remaining 20% as the test set.

For model training, the same objective function, optimizer, and hyperparameters were used. The objective function is the cross-entropy loss function, and the optimizer is the Adam optimization algorithm. The initial learning rate of the model was 0.001, and we used a step-by-step learning rate decay strategy, where the learning rate was reduced to 50% of its original value every 20 epochs. A total of 200 epochs were trained.

### 4.1. MFAREn Feature Extraction Effect

**Figure 6:**
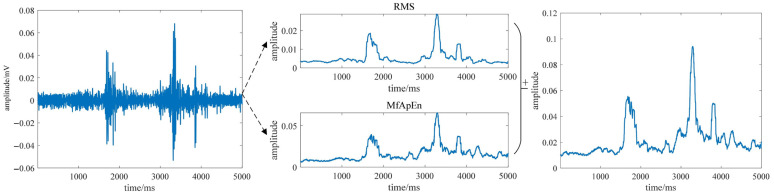
Entropy feature extraction process diagram.

The adjustable parameters of MFAREn are mainly *m* and τ. In the experiment, most signals can complete the feature extraction of sEMG signals with three scale factors and two embedding dimensions. To ensure consistency without affecting the experimental results, the same parameters were used for the feature extraction of all the sEMG signals (m = 2, τ = 3). This choice was made considering both the computational efficiency and the nature of the sEMG signal. When m and r were set greater than 3, the resulting entropy values showed large deviations between the resting and active states, which may distort the actual muscle activity patterns. Therefore, to balance the computational complexity and ensure that the entropy values accurately reflected the muscle activation, we selected m = 2 and τ = 3. The results are shown in Figure 6, using the VM channel sEMG signals in the level walking mode as an example.

As can be seen from Figure 6, after multi-scale fuzzy approximate entropy and RMS feature extraction, the sEMG signals can be preliminarily distinguished between the resting and active states. In the resting segments, the RMS-based feature values exhibited a smooth overall curve, reflecting the general trend of sEMG signals. In contrast, multi-scale fuzzy approximate entropy feature values showed small-amplitude fluctuations, capturing the nonlinear characteristics of sEMG signals. By combining both features, as shown in the rightmost part of Figure 6, the active and resting states of sEMG signals can be clearly distinguished throughout the gait cycle, effectively capturing both nonlinear details and the overall signal variations.

**Figure 7:**
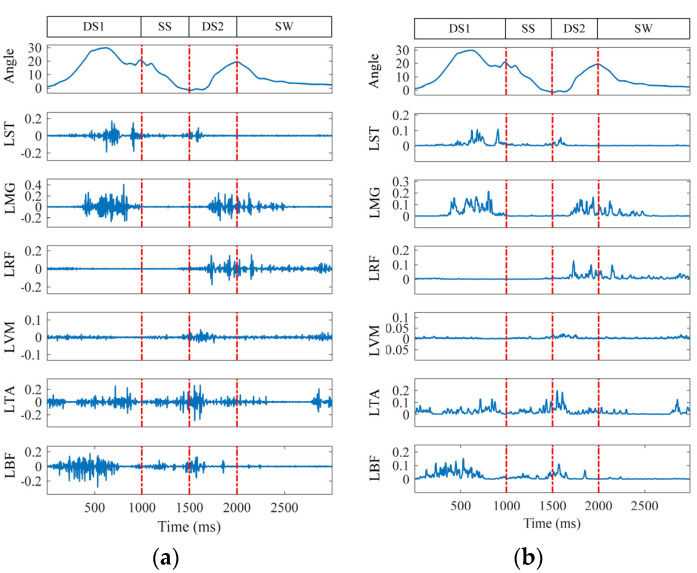
Graph of feature extraction results. (**a**) Raw gait data. (**b**) Entropy characterization of gait data.

The proposed method exhibits significant feature extraction performance across multiple channels. As shown in Figure 7, the left leg’s six-channel sEMG signals in the level walking mode are taken as an example.

As shown in Figure 7, compared to the raw sEMG signals, the sEMG signals processed via MFAREn can effectively reflect the muscle resting and active states. As illustrated in Figure 7b, during the first phase, the sEMG signals of the ST, MG, TA, and BF muscles are in an active state, showing significant amplitude changes after feature extraction. Meanwhile, the sEMG signals of the RF and VM muscles remain in a resting state, with their feature amplitudes approximating to zero. This indicates that the feature extraction method can accurately capture muscle contractions and activity.

### 4.2. EMACNN Ablation Experiments

**Figure 8:**
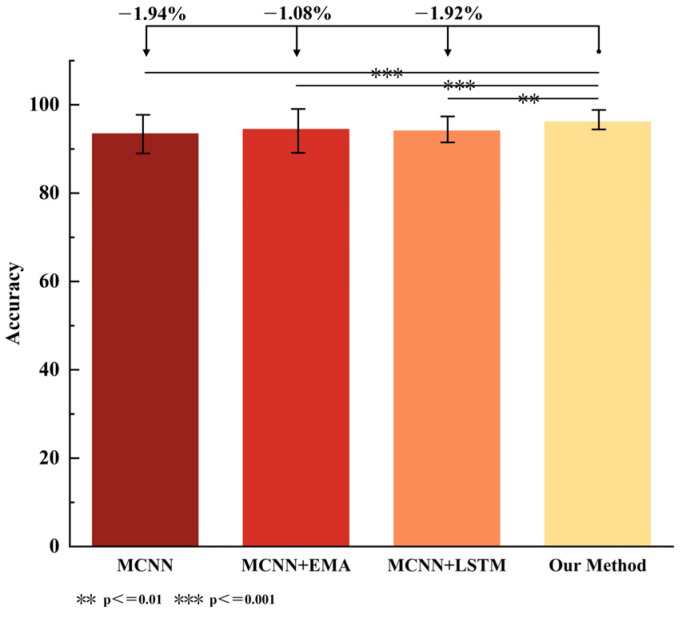
Comparative results of ablation experiments.

In this study, a multi-channel CNN was used as the baseline, with the corresponding improvements denoted by numerical indicators. An ablation experiment was conducted using level walking data (raw data without entropy feature extraction) to independently verify the effectiveness of the neural network. The recognition accuracy of the model was measured by reference to Acc (Accuracy), Pre (Precision), Re (Recall), and F1-score. The real-time recognition performance of the model was evaluated by reference to T (average time consumption), and the computational complexity of the model was assessed via Param (parameter memory). The results are shown in Table 1 and Figure 8. The improvements in this paper include: constructing a multi-channel CNN (MCNN) based on the CNN network, using the EMA mechanism for multi-scale feature extraction, employing LSTM to extract the temporal features of sEMG signals, and fusing the spatial and temporal features of the EMG signals.

From Table 1, it can be seen that adding the EMA module to the baseline can effectively improve the recognition accuracy. Specifically, the accuracy was improved by 0.86%, precision by 0.86%, recall by 0.87%, and F1-score by 0.87%. Analyzing the experimental data, the EMA, as a plug-and-play network module, achieved better performance improvement without increasing the complexity of network computations. Similarly, using LSTM to extract the timing features on the basis of the baseline also improved the accuracy. After fusing the LSTM and EMA modules, the recognition accuracy was further improved compared to the baseline. At this point, the model’s accuracy improved by 1.94%, precision by 1.88%, recall by 1.93%, and F1-score by 1.92%. The baseline model demonstrated the best performance in real-time capability (time consumption) and computational efficiency (parameter count), but achieved the lowest recognition accuracy (Acc = 94.83%). In contrast, although the EMACNN constructed by incorporating EMA and LSTM significantly improved the accuracy (Acc = 96.77%), it sacrificed some real-time capability, with an increase of 0.8 ms in time consumption and 0.15 M in parameter count. Therefore, the model proposed in this paper can effectively improve the recognition accuracy and has good real-time performance, which is crucial for gait phase recognition. Through one-way ANOVA, it can be concluded that there are significant differences in the accuracy between the combinations of modules and the method proposed in this paper. In the future, the phase recognition of gait motion data in multiple scenes will be carried out based on the EMACNN model proposed in this paper.

### 4.3. MFAREn-EMACNN Classification Performance Evaluation

To verify the effectiveness of the EMACNN model designed in this paper for feature extraction, the test set data were input into the EMACNN model. Subsequently, the feature maps of each layer were extracted and converted into feature vectors.

**Figure 9:**
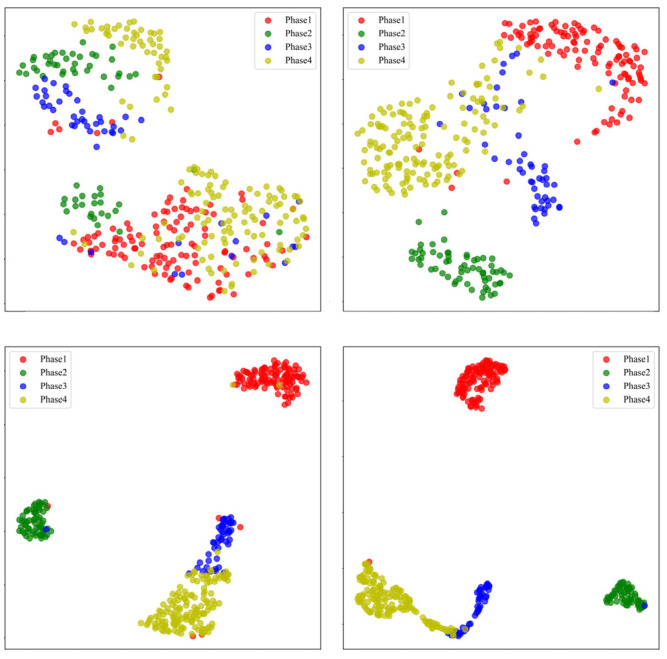
EMACNN feature maps for each layer of UMAP downscaling visualization results.

These feature vectors were then downscaled to two dimensions using the uniform manifold approximation and projection (UMAP) method for visualization, as shown in Figure 9. The four colors correspond to the four gait phases.

As shown in Figure 9, after the first layer of the EMACNN model processing, the distribution of the feature samples for the four gait phases was relatively confusing. However, after the second layer of calculation, the feature samples of each phase began to show a trend of separation. By the third layer of calculation, some of the samples were better separated, but there was still some confusion among the others. Ultimately, after the fully connected layer, the four types of samples were clearly separated. This indicates that the EMACNN model designed in this paper can effectively perform deeper gait phase differentiation for sEMG signals after MFAREn feature extraction.

### 4.4. Comparison with the State-of-the-Art Methods

**Figure 10:**
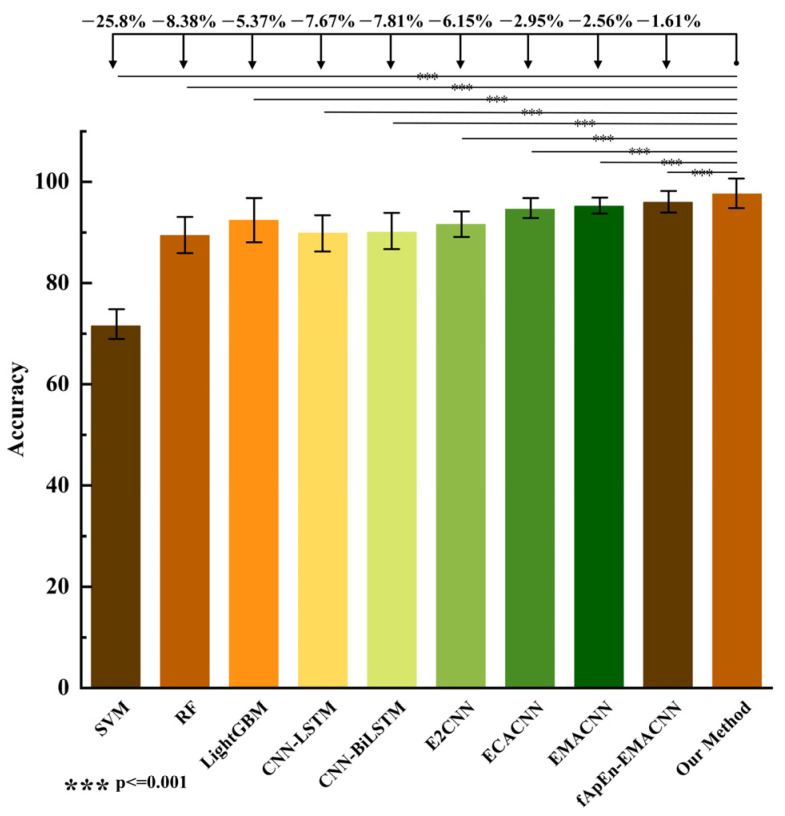
Comparison effect diagram of various methods.

To further validate the effectiveness and generalization ability of the proposed MFAREn-EMACNN recognition method, we conducted multiple comparative experiments using level walking data. In these experiments, the training and test datasets for all level walking data were split in a 3:1 ratio. The proposed method was compared and analyzed with the other methods based on a five-fold cross-validation approach, such as RF, SVM, CNN-LSTM [24], CNN-BiLSTM [37], and E2CNN [38]. The accuracy of the various recognition methods is shown in Table 2, and the significance comparison results are presented in Figure 10.

From Table 2, it can be seen that the accuracy of the machine learning classification models is lower than that of the neural network classification models. Specifically, the evaluation indexes of SVM and RF are below 90%, while the accuracy of the LightGBM classification model is better than that of SVM, RF, CNN-LSTM, CNN-BiLSTM, and E2CNN. The classification accuracy of the ECACNN and EMACNN models is superior to that of most of the models due to the adoption of the multi-channel attention mechanism, which verifies its effectiveness. In addition, traditional machine learning methods offer the advantage of lightweight computation with low parameter counts, but generally suffer from lower recognition accuracy. Deep learning approaches improve the accuracy by introducing more complex network structures and larger model sizes, often at the expense of increased inference time. The method proposed in this study combines MFAREn feature extraction with the EMACNN classifier. Although MFAREn introduces some computational overhead, the overall average recognition time remains 11.26 ms: slightly higher than some other methods but still within the acceptable range for real-time applications. Notably, the proposed method achieves high accuracy with only 0.62 M parameters, significantly fewer than those required by more complex models such as CNN-LSTM (2.18 M) and CNN-BiLSTM (4.30 M). While the parameter count is slightly higher than that of LightGBM (0.02 M), our method strikes an effective balance between the model’s complexity, computational efficiency, and performance. This demonstrates its strong potential for practical deployment and real-time use.

As shown in Figure 10, the proposed method is significantly different from several methods (*p* < 0.001), as determined via one-way ANOVA. Compared to the accuracy indexes of the previous nine classification models, the MFAREn-EMACNN model proposed in this paper exhibits the highest classification accuracy across multiple motion modes. Taking flat walking data as an example, our method improved the accuracy by 2.95% and 2.56%, precision by 3.04% and 2.45%, recall by 2.92% and 2.53%, and F1-score by 3.02% and 2.57%, compared to the ECACNN and EMACNN models. This demonstrates that the MFAREn-EMACNN model achieves higher classification accuracy than the other classification models. The method presented in this paper further enhances the classification accuracy through feature extraction based on original sEMG signals. Therefore, the combination of MFAREn features with the EMACNN neural network enables high-precision gait phase recognition.

### 4.5. Experimental Validation of Multi-Scene Gait Phase Recognition

**Figure 11:**
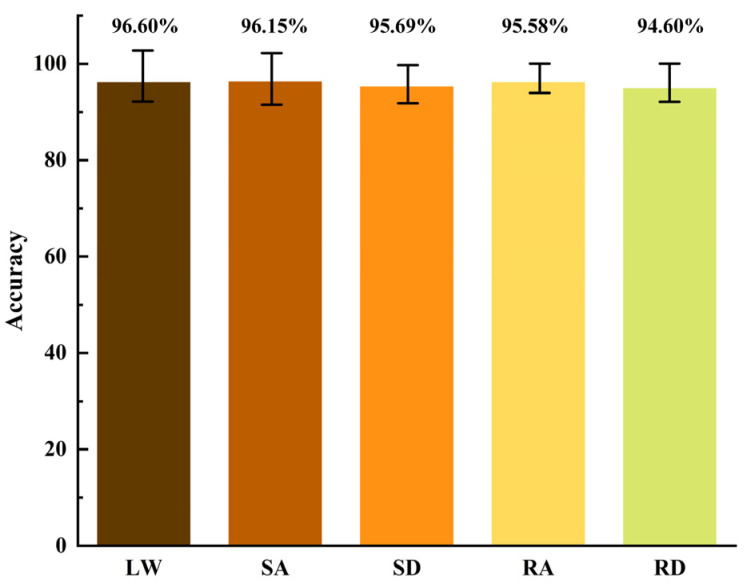
Recognition accuracy in different motion modes.

By combining the MFAREn and EMACNN models, this study tested their effectiveness and generalization based on datasets for level walking (LW), stairs ascending (SA), stairs descending (SD), ramp ascending (RA), and ramp descending (RD). The recognition accuracy under each dataset is presented in Table 3 and Figure 11.

As can be seen from Table 3, the method proposed in this paper can achieve gait phase recognition under five different motion scenarios. Among these, the method is particularly effective for gait phase recognition during level walking and stairs ascending, with an average accuracy of over 96% for three groups of new subjects. However, the accuracy for gait phase recognition during stairs descending and ramp descending were still above 94%. The reduction in the accuracy for descending stairs and slopes can be attributed to potential muscle jitter, which may compromise the integrity of the sEMG signal compared to level walking and ascending stairs.

**Figure 12:**
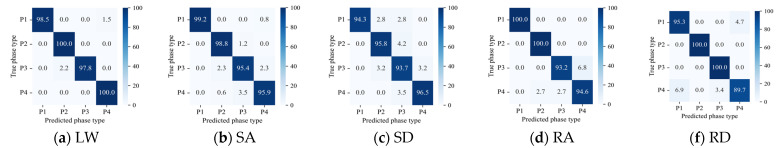
Confusion matrix in different motion modes.

The recognition results of the MFAREn-EMACNN model on the test set for level walking (LW), stairs ascending (SA), stairs descending (SD), ramp ascending (RA), and ramp descending (RD) are shown in Figure 12. P1 represents the first double support phase (DS1), P2 represents the single support phase (SS), P3 represents the second double support phase (DS2), and P4 represents the swing phase (SW).

As shown in Figure 12, the recognition accuracy for the majority of categories is above 97%, with some phases achieving 100% accuracy. This indicates that the method proposed in this paper can accurately identify four gait phases, such as the second and fourth phases in the level walking mode, the first and second phases in the ascending mode, and the second and third phases in the descending mode. This suggests that there are significant differences in the muscle activation across these movement modes, making it easier to distinguish them using the sEMG signals. However, the recognition accuracy for the third and fourth phases in the ascending mode and the fourth phase in the descending mode is below 95%. This may be due to the similar muscle activation patterns between adjacent phases in these motion modes, and the rotation of the sEMG features by the EMACNN neural network may make it more difficult to distinguish these phases.

## 5. Discussion

In the gait phase of common human movement patterns, there is a similarity in muscle force generation pattern. Since the sEMG signals are susceptible to noise interference, gait phase recognition based on the sEMG signals poses difficulties. In this paper, we propose a high-precision hybrid model for gait phase recognition in continuous lower limb movement. Although this model is more complex than partially categorized models, each feature extraction and enhancement module ultimately improves the accuracy of the gait phase recognition during continuous motion.

MFAREn feature extraction improves fuzzy approximate entropy using the overlapping sliding window method and multiscale approach to extract sEMG entropy feature information, taking into account the nonlinear characteristics of the sEMG signal. From the results in Table 2, it can be seen that using entropy features improves the model’s accuracy by 2.56%, indicating that the signal quality input into the recognition model has been enhanced. MFAREn, through fine-grained analysis at different scales, provides a richer and more accurate signal description, which can smooth out random fluctuations in the sEMG data to a certain extent, thus improving the ability to capture the true structure of the signal.

The mapping from the sEMG signals to gait phases is highly nonlinear, making neural networks the mainstream algorithm for solving the sEMG-based gait phase decoding problem. The EMA not only avoids dimensionality reduction but also captures the sEMG features at a larger scale through convolution, enhancing the overall network’s reasoning capability. In this paper, we further incorporate LSTM to extract the temporal features of the sEMG signals and aggregate the output feature maps from multiple channel sub-networks. The results from the ablation experiments show that the aggregation of these modules improves the classification accuracy of the CNN network. This network contributes to the precise recognition of gait phases in continuous lower limb movement patterns.

The proposed method achieves an accuracy of over 95% in recognizing the gait phases of five common movement patterns in daily life, with the recognition accuracy for level walking, stairs ascending, and ramp ascending exceeding 97%. However, due to the similarity of the sEMG signals between adjacent phases in some movement patterns, combined with the inherent invariance of convolutional neural networks to rotation and flipping, misclassifications occurred in phases three and four of ramp ascending, as well as phases one and four of ramp descending. Nonetheless, the method exhibits superior performance in specific gait phases of certain movement patterns. For example, it achieves high recognition accuracy in the second and fourth gait phases of level walking, the first and second gait phases of stair ascent, the first and second gait phases of stair descent, and the second and third gait phases of ramp descent. Additionally, muscle jitter during stairs descending and ramp descending may cause sensor position shifts, which in turn interfere with sEMG signal acquisition and ultimately result in a recognition accuracy below 96% (though still maintaining a precision level of 94%). Future work in this study will focus on addressing the impact of sensor placement variations on the model performance—a research point of significant practical importance, as the displacement of wearable devices may lead to shifts in signal features. We will further optimize the model’s adaptability to sensor position changes to enhance the recognition stability in complex scenarios.

Compared with the related studies in Table 4, the proposed method achieves a high recognition accuracy of 95.72% using only sEMG signals, without relying on multimodal sensors. In terms of accuracy, it is comparable to approaches using multiple sensors. Meanwhile, the model supports multi-task recognition, demonstrating good generalization and real-time performance (average time: 12.59 ms), making it more suitable for continuous gait phase recognition in wearable systems.

The aim of this study was to first establish a robust baseline method using healthy subjects to verify the feasibility, accuracy, and real-time performance of the proposed model under controlled conditions. However, it is worth noting that the recognition performance of the proposed method tends to decline for obese individuals. For instance, Subject 4 in this study had a BMI of 30.14, and a decrease in classification accuracy was observed across all the gait phase categories. Therefore, future research will expand the subject pool to include individuals of different age groups, people with motor impairments, and those with obesity, in order to enhance the model’s generalizability and robustness. These issues will be the key focus areas for future research.

## 6. Conclusions

In order to solve the problem of rapid identification of human lower limb gait phases, this paper proposes a method based on the combination of multi-scale fuzzy approximate entropy and EMACNN. Firstly, MFAREn is used for the feature extraction of sEMG, which can reduce the computational dimension of the signal while extracting the nonlinear and energy features of the electromyographic signals. Then, a high-efficiency multi-channel attention convolutional neural network model, EMACNN, is constructed for feature reinforcement and classification recognition of signal features.

To better distinguish the gait phases in the sEMG signals, we propose a new metric, MFAREn. This feature combines the nonlinear complexity and energy magnitude of the sEMG signals, thereby improving the classification performance of motion analysis. Additionally, the muscle features obtained through this method can retain the subtle variations in nonlinear data trends.

To achieve a higher recognition accuracy, this paper constructs a multidimensional deep neural network consisting of multiscale convolution, batch normalization, efficient multi-scale attention, pooling, and full connectivity. This network can achieve the effective recognition of feature components, and the classification results of the integrated sEMG features solve the problem of the sEMG features being easily confused.

Based on the experimental validation of sEMG data collected from five designed experimental scenarios, the proposed method in this paper is compared horizontally with a variety of mainstream classification methods. The proposed method is significantly different from several methods (*p* < 0.001). The experimental results show that the average recognition accuracy of the proposed method is 95.72%, with 96.60% for level walking, 96.15% for stairs ascending, 95.69% for stairs descending, 95.58% for ramp ascending, and 94.60% for ramp descending. Taken together, the proposed method can complete the gait phase recognition in multiple scenarios and outperforms the comparative methods in terms of accuracy, illustrating the effectiveness of the method. In the future, modular integration and more scene validation should be considered to make the method more universal.

## Figures and Tables

**Table 1:** Results of ablation experiments of EMACNN.

Experimental Group	Modeling Approach		Acc/%	Pre/%	Re/%	F1/%	T/ms	Param/M
	EMA	LSTM						
1	MCNN (baseline)		94.83	94.84	94.82	94.84	3.42	0.47
2	√		95.69	95.70	95.69	95.71	3.58	0.50
3		√	94.85	94.86	94.84	94.85	4.02	0.52
4	√	√	96.77	96.72	96.75	96.76	4.22	0.62

**Table 2:** Validation based on data from level walking.

Methods	Acc/%	Pre/%	Re/%	F1/%	T/ms	Para/M
SVM	72.04 ± 0.43	70.45 ± 0.42	72.04 ± 0.42	70.48 ± 0.42	0.86	0.24
RF	89.46 ± 0.48	89.42 ± 0.47	89.46 ± 0.47	89.40 ± 0.48	2.52	0.25
LightGBM	92.47 ± 0.72	92.67 ± 0.72	92.47 ± 0.73	92.52 ± 0.72	0.06	0.02
CNN-LSTM	90.17 ± 0.46	90.22 ± 0.46	90.20 ± 0.45	90.51 ± 0.46	3.20	2.18
CNN-BiLSTM	90.03 ± 0.45	90.21 ± 0.45	90.02 ± 0.44	90.30 ± 0.45	3.40	4.30
E2CNN	91.69 ± 0.24	91.76 ± 0.23	91.69 ± 0.25	91.70 ± 0.24	1.29	0.29
ECACNN	94.89 ± 0.18	94.76 ± 0.18	94.89 ± 0.17	94.82 ± 0.18	11.37	0.48
EMACNN	95.28 ± 0.14	95.35 ± 0.14	95.28 ± 0.14	95.27 ± 0.15	4.22	0.50
fApEn-EMACNN	96.23 ± 0.32	96.22 ± 0.35	96.23 ± 0.35	96.21 ± 0.31	11.78	0.62
Our method	97.84 ± 0.42	97.80 ± 0.42	97.81 ± 0.41	97.84 ± 0.42	11.26	0.62

**Table 3:** Classification accuracy in different motion modes.

Motion Mode	Subject	Age	Acc/%	Pre/%	Re/%	F1/%	T/ms
LW	No.1	20	97.19 ± 0.16	97.13 ± 0.18	97.19 ± 0.16	97.11 ± 0.17	10.27
	No.2	22	97.33 ± 0.28	97.37 ± 0.27	97.38 ± 0.27	97.37 ± 0.28	13.72
	No.3	25	97.53 ± 0.43	97.56 ± 0.43	97.53 ± 0.42	97.54 ± 0.42	14.58
	No.4	27	94.37 ± 0.56	94.43 ± 0.55	94.37 ± 0.56	94.35 ± 0.55	13.89
	No.5	28	96.60 ± 0.66	96.74 ± 0.66	96.60 ± 0.66	96.59 ± 0.65	12.63
SA	No.1	20	97.07 ± 0.19	97.09 ± 0.19	97.07 ± 0.18	97.07 ± 0.19	11.52
	No.2	22	97.45 ± 0.23	97.46 ± 0.25	97.42 ± 0.23	97.44 ± 0.25	10.23
	No.3	25	96.80 ± 0.52	96.80 ± 0.53	96.77 ± 0.53	96.78 ± 0.52	12.38
	No.4	27	94.10 ± 0.46	94.16 ± 0.52	94.10 ± 0.46	94.09 ± 0.48	13.52
	No.5	28	95.32 ± 1.11	95.59 ± 0.94	95.32 ± 1.11	95.25 ± 1.15	13.66
SD	No.1	20	96.09 ± 0.22	96.08 ± 0.22	96.09 ± 0.22	96.07 ± 0.23	12.27
	No.2	22	96.80 ± 0.25	96.87 ± 0.25	96.80 ± 0.27	96.81 ± 0.25	12.57
	No.3	25	96.21 ± 0.58	96.21 ± 0.54	96.24 ± 0.58	96.22 ± 0.58	13.83
	No.4	27	93.89 ± 0.80	93.92 ± 0.86	93.89 ± 0.80	93.86 ± 0.85	13.05
	No.5	28	95.44 ± 0.39	95.48 ± 0.38	95.44 ± 0.39	94.45 ± 0.40	13.82
RA	No.1	20	97.57 ± 0.23	97.64 ± 0.24	97.57 ± 0.23	97.58 ± 0.24	11.92
	No.2	22	97.11 ± 0.33	97.15 ± 0.31	97.11 ± 0.33	97.09 ± 0.33	12.03
	No.3	25	96.38 ± 0.50	96.39 ± 0.49	96.38 ± 0.50	96.40 ± 0.51	12.38
	No.4	27	91.47 ± 1.43	91.65 ± 1.37	91.47 ± 1.43	91.44 ± 1.38	12.08
	No.5	28	95.87 ± 0.53	95.96 ± 0.45	95.87 ± 0.53	95.89 ± 0.51	12.67
RD	No.1	20	95.11 ± 0.36	95.14 ± 0.37	95.10 ± 0.36	95.11 ± 0.37	11.21
	No.2	22	95.38 ± 0.59	95.36 ± 0.61	95.37 ± 0.59	95.41 ± 0.61	12.14
	No.3	25	96.31 ± 0.83	96.32 ± 0.87	96.31 ± 0.83	96.33 ± 0.84	11.67
	No.4	27	92.11 ± 0.94	92.39 ± 0.87	92.11 ± 0.94	92.10 ± 0.90	12.57
	No.5	28	94.11 ± 1.46	94.61 ± 1.28	94.11 ± 1.46	94.03 ± 1.64	12.56

**Table 4:** Comparison with related research.

Ref.	Number of Tasks	Number of Phase	Sensors	Method	Performance
[12]	1	4	IMU	SBLSTM	Acc: 94%
[10]	1	5	EMG + IMU	DCNN-SVM	Acc: 96.00%
[23]	1	4	IMU + pressure sensor	NHMM	Acc: 94.7%
[24]	1	5	IMU + pressure sensor	CNN-PCA-LSTM	Acc: 97.91%
This work	5	4	sEMG	MFAREn-EMACNN	Acc: 95.72%T: 12.59 ms

## Data Availability

The data presented in this study are available on request from the corresponding author. The data are not publicly available due to privacy.
